# Arachidonic acid inhibit granulosa cell function by affecting metabolic function of liver in brown adipose transplantation rats

**DOI:** 10.1186/s13048-024-01374-8

**Published:** 2024-02-19

**Authors:** Yan Yan, Fangfang Di, Ruoxi Zhang, Liwen Song, Runjie Zhang, Jin Qiu

**Affiliations:** Obstetrics and Gynecology Department, Tongren Hospital, Shanghai Jiao Tong University School of Medicine, No.1111, XianXia Road, Shanghai, 200336 China

**Keywords:** PCOS, BAT, Granulosa cells, Arachidonic acid, Metabolites, Liver factor

## Abstract

**Background:**

Polycystic ovary syndrome (PCOS) is a gynecological endocrine disease and could be considered a metabolic disease because it is often accompanied by obesity and insulin resistance. Brown adipose tissue (BAT) transplantation has been shown to be effective in treating PCOS rats.

**Results:**

The study demonstrated that BAT successfully recovered the reproductive and metabolic phenotype of PCOS rats. The disorder estrous cycle, abnormal hyperglycemia and the expression of liver factors were improved. Differentially expressed metabolites were analyzed, among them, arachidonic acid may play a role in inhibiting cell proliferation, enhancing oxidative stress reaction, promoting estrogen expression, and reducing progesterone level in KGN cells.

**Conclusion:**

Our findings suggest that BAT transplantation may be a therapeutic strategy for PCOS by changing the expression of some cytokines and metabolites. Differentially expressed metabolites might be crucially important for the pathogenesis of PCOS.

## Background

Polycystic ovary syndrome (PCOS) is the most common endocrine metabolic disease in women of childbearing age and the main cause of anovulatory infertility [1]. The global PCOS incidence rate increased 1.45% over the period 1990–2017 [2]. According to the 2003 Rotterdam standard, there are 3 main manifestations: clinical or biochemical androgen excess, rare ovulation or anovulation and morphological changes of polycystic ovary [3]. In addition, due to abnormal metabolic function, most PCOS patients will show metabolic syndrome, such as obesity, dyslipidemia, hypertension, hyperinsulinemia and dysglycemia [4], the risk of insulin resistance (IR) and type 2 diabetes mellitus are significantly increased in patients with PCOS [5].

It has been found that brown adipose tissue (BAT) is closely related to PCOS [6]. BAT activity is significantly decreased in PCOS patients, and has beneficial effects in animal models of PCOS [7]. BAT use glucose and fatty acids as fuel to convert the electrochemical gradient of cell respiration into heat to maintain body temperature through thermogenesis in small mammals and neonates, which is a high-energy tissue with unique expression of uncoupling protein-1 (UCP-1) [8]. BAT transplantation could reverse obesity and insulin resistance, significantly increased insulin sensitivity and eventually ameliorated hyperandrogenism, acyclicity and infertility [9].

Searching for the mechanisms of PCOS pathophysiology has become a crucial aspect of research performed in the last decades. In recent years, many studies have proved that liver plays an important role in the pathogenesis of PCOS, the incidence of nonalcoholic fatty liver disease in PCOS patients is significantly higher than that in the general population, and disorder of glucose metabolism could also promote the occurrence and development of liver damage [10, 11]. IR is implicated as a major player in the pathogenesis of hepatocyte injury [12]. The expression level of liver factors may be related to compensatory adjustment for confrontation of dyslipidemia in PCOS [13]. Among the liver factors, carbohydrate-response element-binding protein regulates the transcription of glucose and lipid metabolism genes, hepatic fibroblast growth factor 21 (FGF21) contributes to the improve glucose and lipid homeostasis [14]. However, the effects of liver factors on ovarian granulosa cells and the relationship between liver factors and PCOS remain unclear.

The application of mass spectrometry technology, such as liquid chromatography tandem mass spectrometry, gas chromatography tandem mass spectrometry, give a promising insight into the research on PCOS [15]. Metabolite-protein interactions control a variety of cellular processes, which means active metabolites may not only serve as biomarkers or be metabolized through several metabolic pathways, leading to the generation of biologically active compounds, but also have many physiological functions and are closely related to the occurrence of several diseases and involved in the pathology of diseases [16]. Ban et al. found that the expression of fatty acid metabolites in follicular fluid of PCOS patients were different from normal women, and 108 lipids were considered as the potential candidate differential metabolites [17]. They The identification of metabolites and their effects are extremely important for the research of PCOS.

Dehydroepiandrosterone (DHEA) is a precursor of androgen synthesis, and its level gets elevated in women suffering from PCOS. A classical way to induce PCOS is by administering DHEA for consecutive days [18]. In our study, we investigated the effects of BAT transplantation, the differential expressed metabolites of BAT transplantation PCOS rats, and the effects of metabolites on ovarian granulosa cells. Our study might provide a novel sight on the and potential therapeutic effects and exploration of mechanism of PCOS.

## Results

### Characteristics of PCOS rat and BAT transplantation rat

**Fig. 1:**
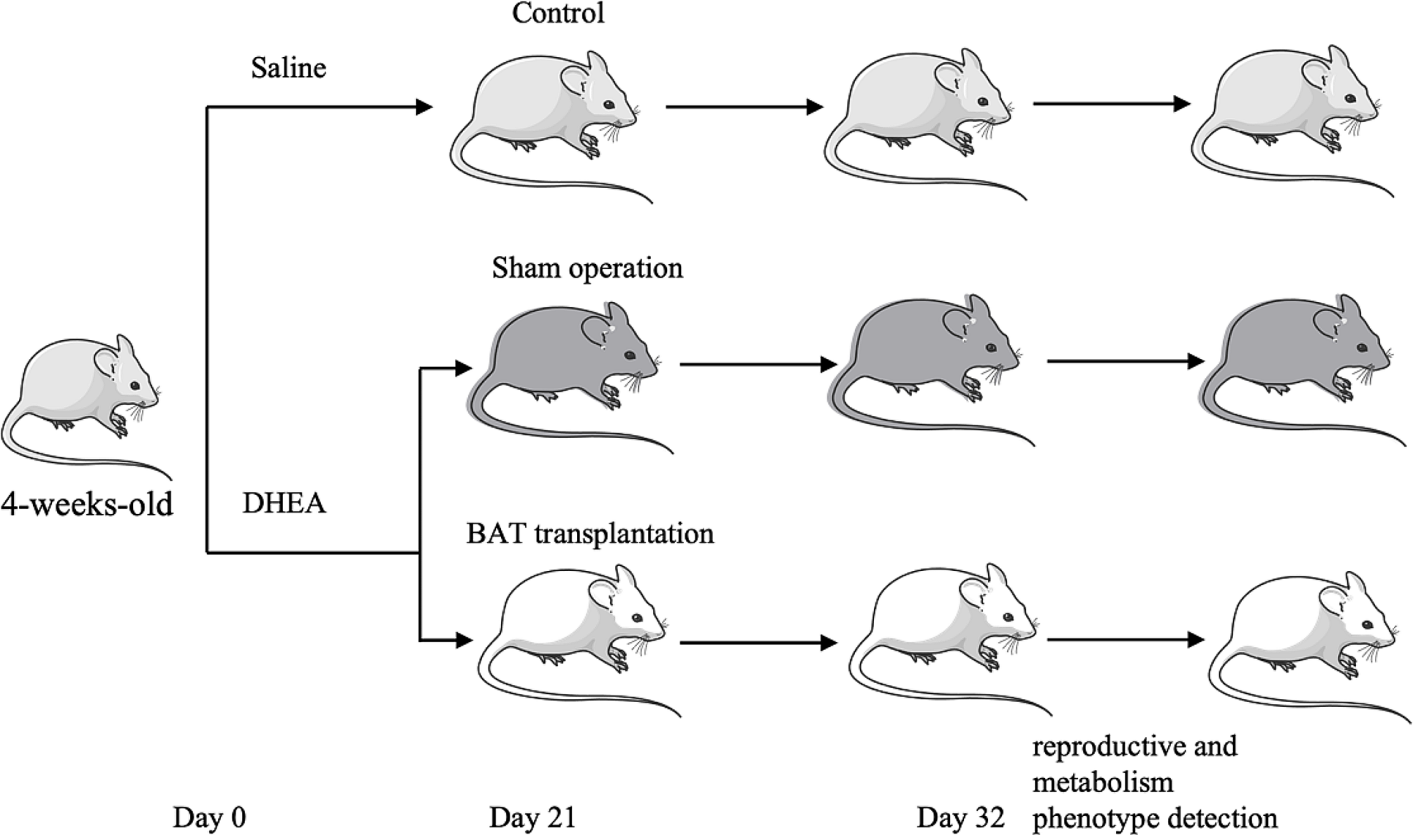
Flow chart of the research. S/D rat were treated with saline and DHEA randomly, and divided into control group and PCOS group. After surgery, PCOS group were divided into sham operation (PCOS + sham) group and BAT transplantation (PCOS + BAT) group. Staining was performed to determine the alteration of ovarian and BAT pathology, estrus cycle and glucometabolic phenotypes observation were conducted. And the liver tissue was collected, we detected the changes of liver factors, performed untargeted metabolomics analysis to search differential expression metabolites. Finally, we investigated the function of those metabolites

We induced PCOS rat by DHEA, then established BAT transplantation model. As shown in Fig. 1. S/D rats were randomly divided into control group and PCOS group and then treated with normal saline or DHEA. Staining was performed to determine the alteration of ovarian and BAT pathology, estrus cycle and glucometabolic phenotypes observation were conducted in the control (CTL) group, sham-operated (PCOS + sham) group and BAT transplantation (PCOS + trans) group. And the liver tissue was collected, we detected the changes of liver factors, performed untargeted metabolomics analysis to search differential expression metabolites. Finally, we investigated the function of those metabolites.

**Fig. 2:**
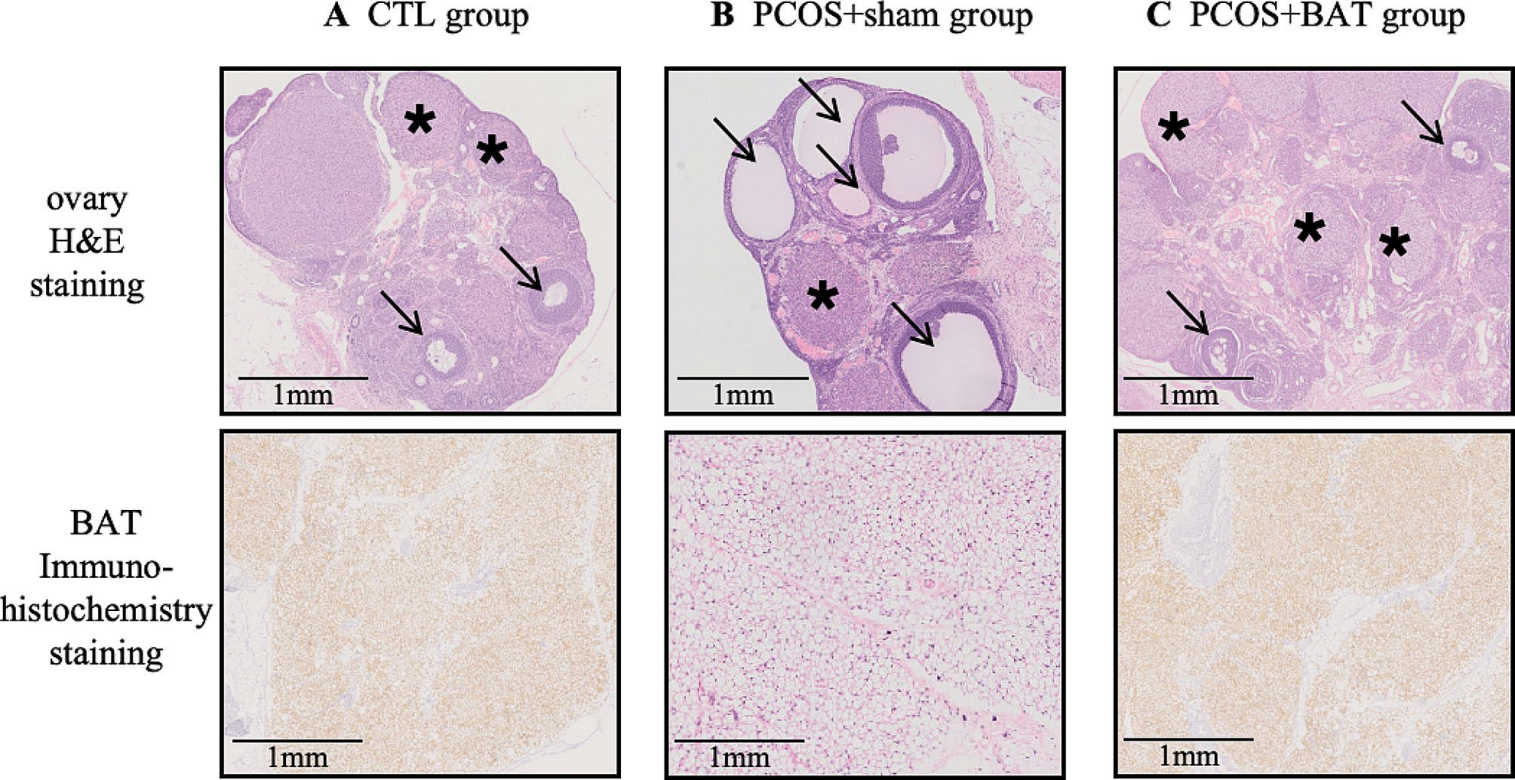
H&E staining and immunohistochemistrical staining results of ovarian tissue slices. The follicular development of ovarian tissues was analyzed. Staining revealed that compared with CTL group and PCOS + BAT group, PCOS + sham group had fewer corpus luteum, more cystic follicles and less UCP-1 positive cells. Cystic follicles were indicated by arrows and corpus luteums were indicated by asterisk

HE staining of ovary tissue and UCP-1 immunohistochemistry were performed to determine if the BAT transplantation exerted normal function. We showed that PCOS + sham group had fewer corpus luteum, more cystic follicles significantly. At same time, stronger UCP-1 signals in the CTL group and PCOS + BAT group than in the PCOS + sham group (Fig. 2).

**Fig. 3:**
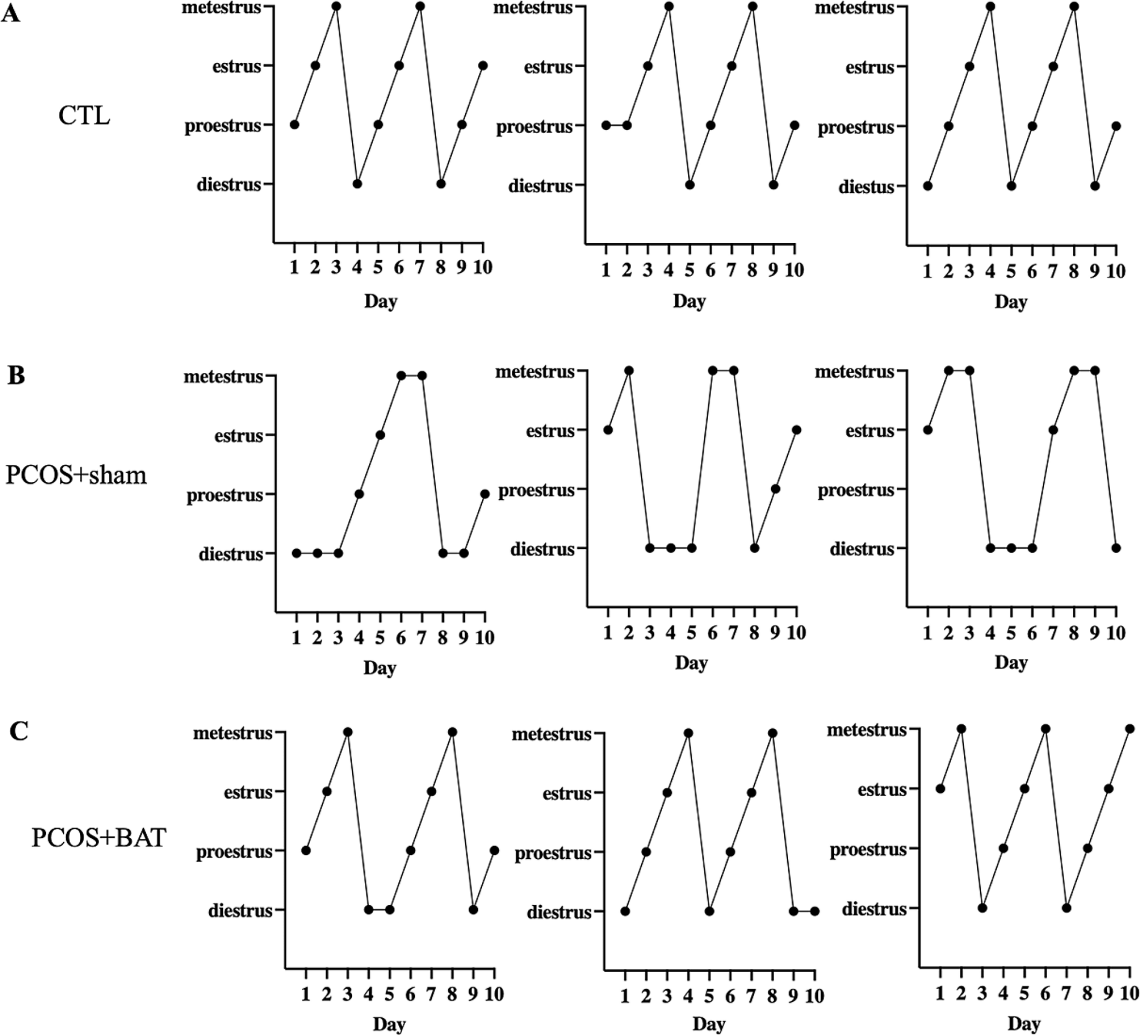
Changes in the estrous cycle. Disordered estrous cycles were observed in the PCOS + sham group, while BAT transplantation reversed the irregular cycles

**Fig. 4:**
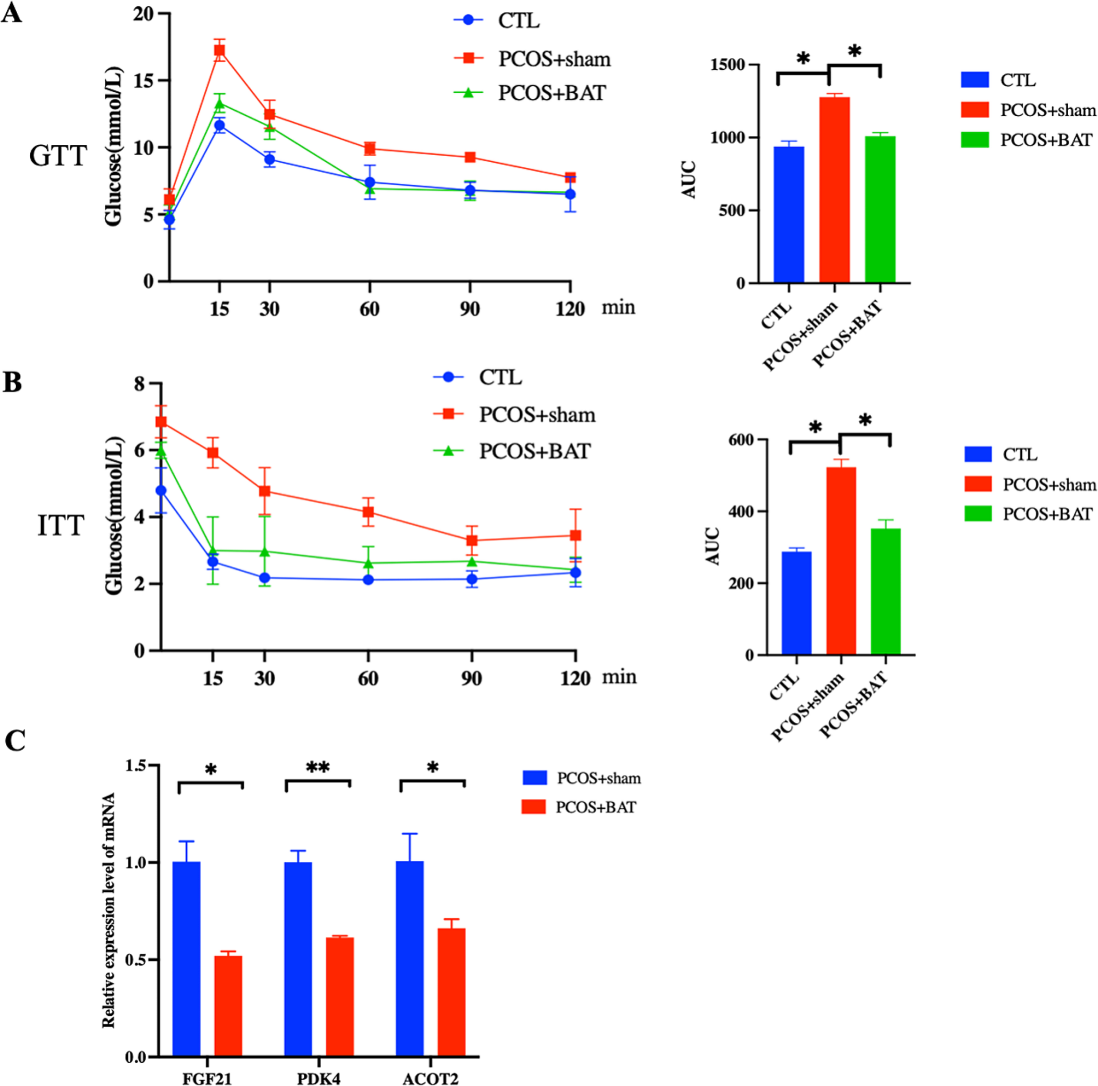
Changes in glucose metabolism and liver factors in rats. The glucose metabolism and liver factors expression of the rat was observed before and after transplantation. BAT transplantation could improve the metabolism phenotype of PCOS. **(A-B)** The level of glucose of PCOS + sham group was significantly higher than that of other groups. **(C)** The expression of liver factors mRNA such as FGF21, PDK4 and ACOT2 were enriched in the PCOS + sham group. * represents P value was less than 0.05

The estrous of the rat changed as well. Disordered estrous cycles were observed in the PCOS + sham group, while BAT transplantation reversed the irregular cycles (Fig. 3). Both GTT and ITT showed that the PCOS + sham group had significantly higher blood glucose levels than the control rat and PCOS + BAT rat (Fig. 4A-B). Our RT-PCR results demonstrated that compared to PCOS + BAT group, the expression of liver factors mRNA such as FGF21, PDK4 and ACOT2 were enriched in the PCOS + sham group (Fig. 4C). Preliminary results revealed that BAT transplantation successfully rescued the glucose metabolism disorder and liver factors back to that of the control group.

### Detection of the metabolic profile in rats

**Fig. 5:**
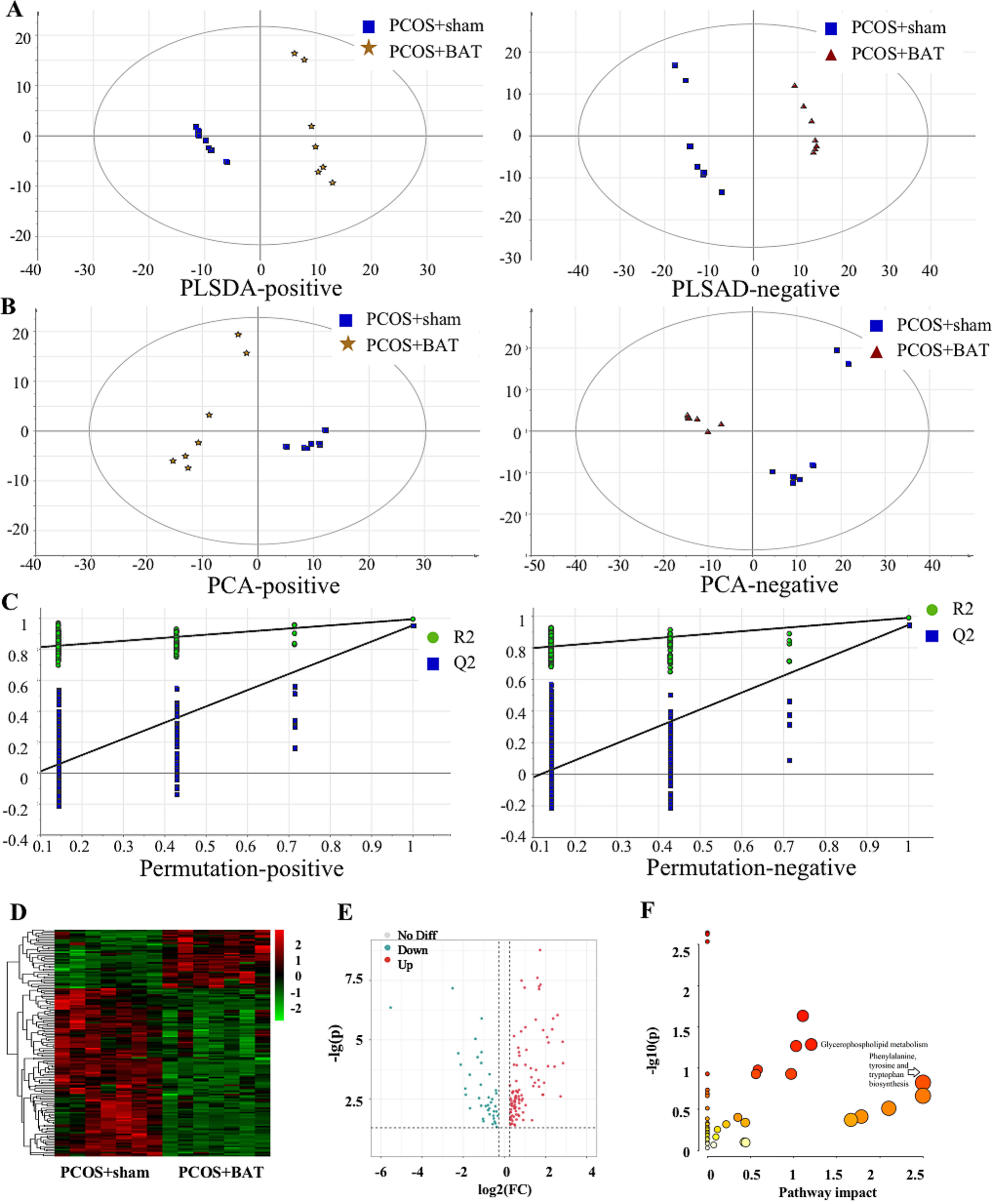
Identified metabolic profile and differentially expressed metabolites in BAT from the PCOS + sham and PCOS + BAT groups. **(A)** PCA showed a distinct metabolic profile between two groups. X-axis and Y-axis represent the first and second principal components respectively. **(B)** Statistical validation with perripening analysis of the corresponding OPLS-DA model of the PCOS + sham and PCOS + BAT group. **(C)** Perripening tests were obtained from LC–MS data of the two groups. The intercept values of the regression line and the Y-axis are R2 and Q2. **(D)** Heatmap of the metabolites that were respectively expressed between PCOS + sham group and PCOS + BAT group. **(E)** Volcano plot of all metabolites expressed in PCOS + sham group and PCOS + BAT group. **(F)** All matched pathways were displayed as circles. The size of the bubble represents the number of metabolites enriched

To study the effect of BAT transplantation on liver tissue metabolism in PCOS rats, the liver tissues were taken from the PCOS + sham and PCOS + BAT rat for metabonomics analyses. Firstly, the results of the PCA analysis revealed the trend of intra-group and inter-group segregation between two groups (Fig. 5A). Figure 5B indicated that the significant differences in metabolites between the two groups since the PCOS + sham group’s plots were far from those of the other group. Besides, a perripening examination of the PLS-DA model was conducted (Fig. 5C).

Enrichment analyses on differential metabolites were performed. The differential metabolites with fold change > 1.2 or < 0.67, VIP > 1 and *P* < 0.05 were clustered and displayed in Fig. 5D-E. Elevated and decreased metabolites were depicted by red and green colors respectively. Moreover, the enriched metabolic pathway clustering analysis demonstrated that differential metabolites were associated with (Fig. 5F). Among all the differentially expressed metabolites between the PCOS + sham and PCOS + BAT groups, the 21 most changed metabolites were listed in Table 1. They mainly belonged to glycerophospholipids and fatty acyls. Among them, 11 metabolites were up-regulated in PCOS + sham group, while 10 metabolites up-regulated. These results indicated significant differences in metabolites between the two groups.

**Table 1:** The most Differentially expressed metabolites

Class	Name	VIP	Fold change
Organooxygen compounds	D-Sedoheptulose 7-phosphate	1.01694	1.40
Organic phosphoric acids and derivatives	Glycerophosphoglycerol	1.73054	1.97
Glycerophospholipids	LysoPC (16:0/0:0)	1.28805	0.64
Glycerophospholipids	LysoPE (0:0/20:5(5Z,8Z,11Z,14Z,17Z))	1.1533	1.59
Glycerophospholipids	LysoPE (0:0/22:2(13Z,16Z))	1.35787	1.78
Glycerophospholipids	LysoPE (0:0/22:5(4Z,7Z,10Z,13Z,16Z))	1.47092	3.16
Fatty Acyls	Palmitoylcarnitin	1.49911	0.61
Glycerophospholipids	PC (19:3(10Z,13Z,16Z)/0:0)	1.37993	1.54
Glycerophospholipids	PE (16:0/0:0)	1.76584	3.33
Glycerophospholipids	PS (20:0/0:0)	1.10867	1.89
Purine nucleosides	Xanthosine	1.21349	1.25
Phenols	3,4-Dihydroxybenzylamine	1.27182	0.46
Steroids and steroid derivatives	3beta,7alpha-Dihydroxy-5-cholestenoate	1.73012	0.17
Fatty Acyls	Arachidonic acid	1.43989	1.81
Fatty Acyls	Docosahexaenoic acid	1.54403	7.21
Benzene and substituted derivatives	Lepidine C	1.26912	1.41
Fatty Acyls	Oleamide	1.11455	1.21
Fatty Acyls	Palmitic acid	1.32063	1.43
Glycerophospholipids	PS (18:1(9Z)/0:0)	1.15168	1.63
Fatty Acyls	Oleoylcarnitin	1.25622	0.53

### Effects of the changed metabolites on KGN cells

Further study on the functions of the changed metabolites, we analyzed docosahexaenoic acid, LysoPC (16:0/0:0), palmitic acid, xanthosine and arachidonic acid (AA). Among all the liposoluble metabolites, we found that only AA has an impact on the function of PCOS granulosa cells, which was highly expressed in PCOS + sham group. KGN cells were pretreated with DHEA to mimic the pathophysiological status of PCOS then adding AA.

**Fig. 6:**
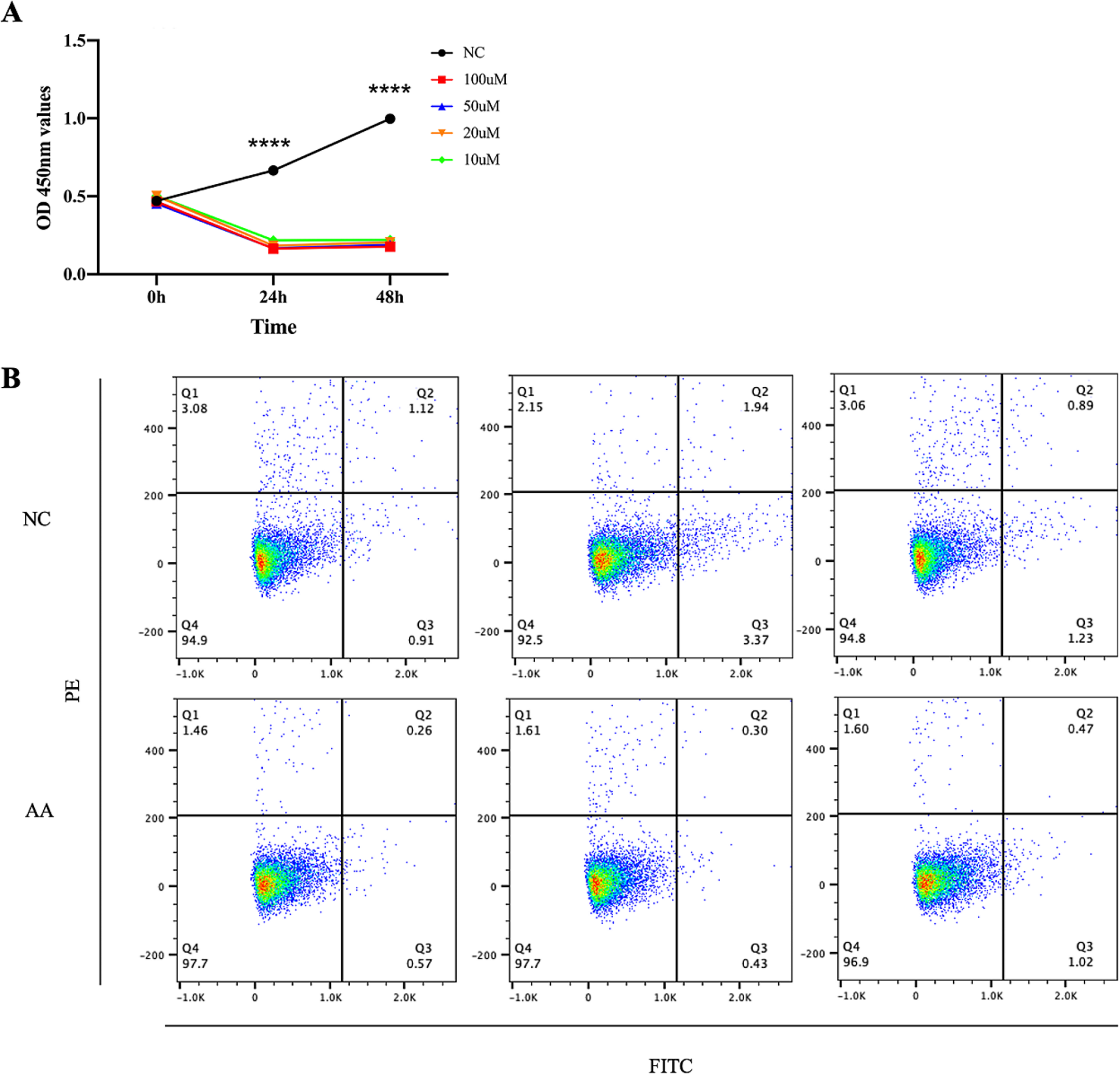
Effects of AA on apoptosis and proliferation of KGN cells. **(A)** AA significantly inhibited the proliferation of KGN cells, regardless of the concentration (**** means *p* < 0.0001). **(B)** The flow cytometry data showed that AA had little effect on cell apoptosis, and there was no statistical difference between CTL group and AA group

**Fig. 7:**
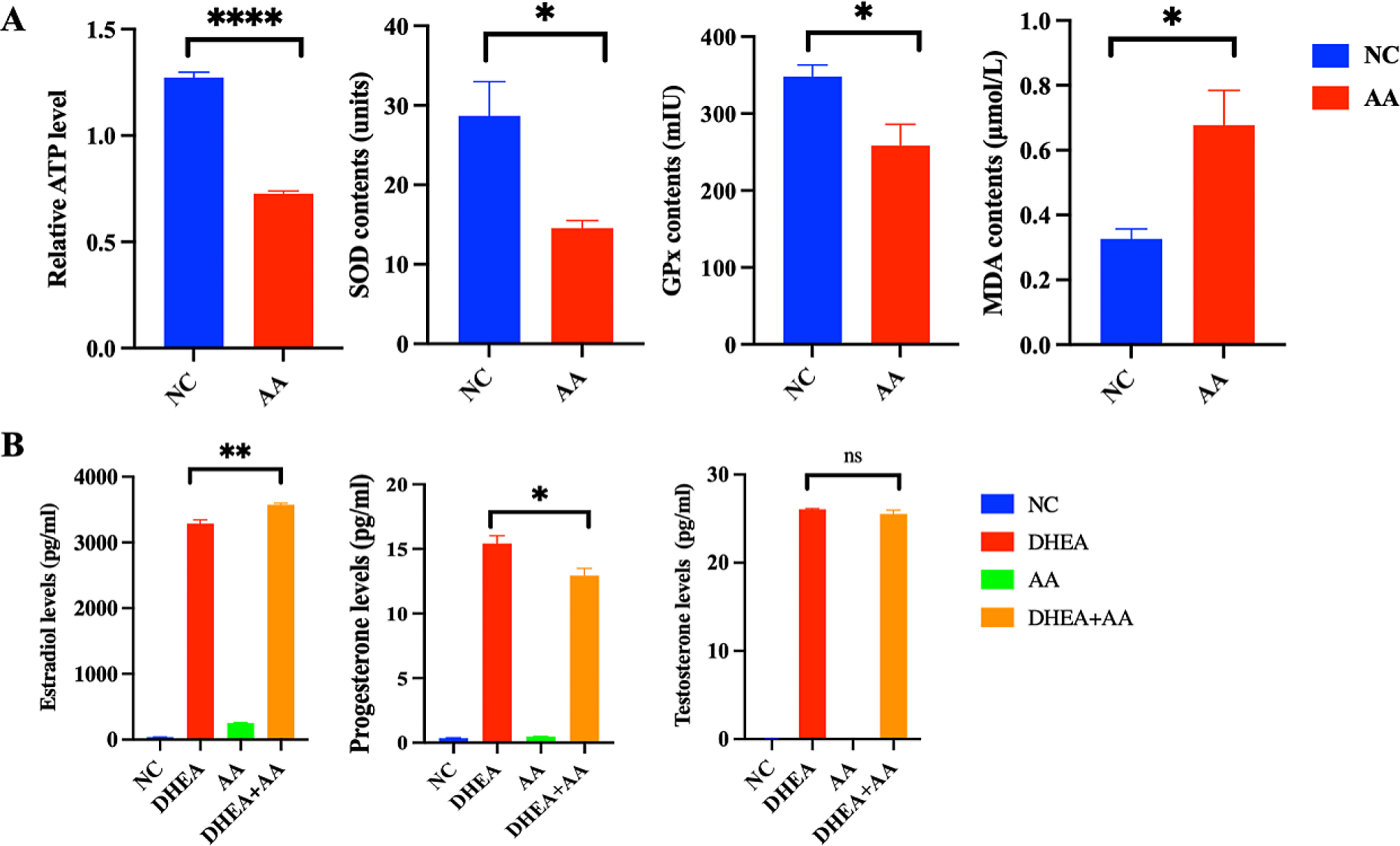
Effects of AA on oxidative stress reaction and hormone secretion in KGN cells **(A)** The NC group had higher levels of ATP, SOD and GPx than AA group. While the level of MDA was lower. **(B)** The estradiol was induced by AA in KGN cells treated by DHEA and AA inhibited progesterone secretion in DHEA-KGN cells. (ns means there was no statistical difference, **** *p* < 0.0001, ** *p* < 0.01, * *p* < 0.05)

CCK-8 assay results indicated that AA significantly inhibited the proliferation of KGN cells, when the concentrations of AA was 10µM, 20µM, 50µM, 100µM respectively. The significance of our findings is illustrated in Fig. 6A. However, the flow cytometry data showed that AA had little effect on cell apoptosis, there was no statistical difference between CTL group and AA group (Fig. 6B). Moreover, AA promoted oxidative stress response of KGN cells, the blank (NC) group had higher levels of ATP, SOD and GPx than AA group. While the level of MDA was lower (Fig. 7A). In addition, the estradiol was markedly induced by AA in KGN cells treated by DHEA and AA inhibited progesterone secretion in DHEA-KGN cells (Fig. 7B).

## Discussion

PCOS is a major severe ovary disorder, BAT contains abundant mitochondria and adipokines and it has been proven to be effective. Here we report that transplantation of BAT reversed the expression of some liver cytokines and metabolites, reproductive and metabolic phenotype of DHEA induced PCOS rats. The researchers observed multiple metabolic improvements after BAT transplantation in a previous study [19]. BAT transplantation significantly decreased the blood glucose and lipid, reversed the pathological changes of liver [20]. As a classical endocrine hormone and an autocrine factor, FGF21 transduces FGF signals to mitogen-activated protein kinase signaling cascades, leads to the recruitment and activation of phosphatidylinositol 3-kinase, then increases glucose uptake in adipose tissue [21]. Human serum FGF21 levels also correlate positively with body mass index, as evidenced by the significantly increased level of serum FGF21 in obese patients and the increased levels of these factors may be a compensatory mechanism that maintains normal glucose and lipid metabolism [13], our results were consistent with Kehinde’s and Huang’s research [22, 23]. Pyruvate dehydrogenase isoenzyme 4 (PDK4) which serves as an adaptor of hepatocyte metabolism, inhibition reprograms glucose and lipid metabolism by enhancing hepatic protein kinase B signaling and activating an adenosine monophosphate-activated protein kinase/forkhead box protein O1/CD36 regulatory axis of lipid [24]. Acyl-CoA thioesterases (ACOTs) hydrolyze fatty acyl-CoA esters, which is mainly enriched in mitochondrial matrix which helps to prevent β-oxidation overload by increasing the availability of CoA [25]. The miR-27b-ACOT2 axis promotes lipid accumulation via its associations with peroxisome proliferators-activated receptors-γ and CCAAT/enhancer binding proteins alpha-α and enhance the hepatic oxidation of fatty acid in liver mitochondria [26], coincide with our RT-PCR results. Overall, FGF21, PDK4 and ACOT2 all play important roles in regulating glucose and lipid metabolism. Due to the profound impact of glucose and lipid metabolism on the function of ovarian granulosa cells, therefore, the expression of these three genes is extremely important for the occurrence and development of PCOS. In our study, we found that after BAT transplantation, the level of FGF21, PDK4 and ACOT2 mRNA was decreased, although the metabolic phenotype of PCOS rats were improved, which means changes of the expression of these genes may be closely associated with the observed metabolism disorder in DHEA-induced rats.

Elucidating the PCOS-related metabolomics have been considered as a key for exploring the mechanisms of PCOS. Metabolomics has proven to be a potential tool in studying the pathophysiology of PCOS. The main abnormalities are associated mainly with the metabolism of lipids, fatty acids, sphingolipids and glycerophospholipids, steroids as well as carbohydrates and amino acids [15]. Fan et al. observed the abnormalities in hormone metabolism and lipid metabolism disorder [27]. The decreased level of phosphocreatinine was observed in PCOS patients, while the contrary trend was shown for LysoPC [28]. In Zou’s study, 35 urine metabolites were found to be significantly different between the PCOS patients and the controls [29]. We executed an untargeted metabonomics analysis, levels of glycerophospholipid and fatty acyls were significantly altered in liver tissue, and among all the 21 most differential expression metabolites, AA has cytotoxicity to KGN cells, which was highly expressed in PCOS + sham group.

AA is one of the most abundant, active and widely distributed polyunsaturated essential fatty acids in the human body, with strong biological activity [30]. It could attenuate cell proliferation, migration and viability [31], for example, in MCF-7 and MDA-MB-231 human breast cancer cell lines, AA inhibited cell proliferation in a dose-dependent manner [32]. AA is also associated with activation of intrinsic apoptosis [33]. However, Liu et al. found that AA and/or their metabolites may enhance tumor growth not only by promoting cell proliferation but also by suppressing apoptosis [34]. We showed that AA significantly inhibited the proliferation of KGN cells, regardless of the concentration but had little effect on cell apoptosis, and there was no statistical difference between CTL group and AA group. Variance results may be related to the inhibition of the different signal transduction pathway involved in different cells.

The AA metabolic network produces a large number of inflammatory mediators, and it has been implicated in inflammatory diseases [35]. After exposure to AA, the activities of ATP, antioxidant enzymes glutathione peroxidase (GPx) and superoxide dismutase (SOD) in KGN cells decreased, while oxidative stress (OS) biomarkers including malondialdehyde (MDA) level were found to increase in KGN cells. Antioxidant defense system, mainly composed of enzymes, such as SOD and GPx. They are widely considered as the key antioxidants in the defense against oxidative stress [36]. And MDA is one of the most important products of membrane lipid peroxidation, and its elevated values are associated with metabolic syndrome and oxidative stress. It is also an important biomarker of oxidative stress and cell damage [37]. In addition, there is a close relationship between OS and inflammation, OS can participate in PCOS pathophysiology as well [38]. Compared with health women, patients with PCOS presented higher circulating concentrations of OS markers [39]. The initial OS induced mitochondrial damage led to the increase of oxidation products and the reduction of ATP production [40]. AA stimulates excessive OS production in cells, which also leads to the reduction of total antioxidant capacity and antioxidant activity of antioxidant enzymes. The blank group had higher levels of ATP, SOD and GPx than AA group, while the level of MDA was lower as an important inflammatory factor. There is a complex relationship between AA and oxidative stress, which is of great significance to the diagnosis and treatment of PCOS.

Changes in hormonal functions are considered a significant factor in developing PCOS in women. Synthesis of estrogen progesterone and testosterone is an important biological function of ovarian granulosa cells, patients with PCOS have high serum levels of androgen and estrogen, leading to failure of ovulation. Therefore, a low level of progesterone in patients with PCOS has been observed [41]. AA plays important roles in human fertility and the course of pathological pregnancies as well [42]. In our research, DHEA was added to the culture medium of KGN cells as a synthetic raw material for steroid hormones. The figure suggested that AA promoted the expression of estrogen and inhibited the secretion of progesterone, but had little effect on the secretion of testosterone of KGN cells. Recent studies have demonstrated that the content of AA are higher in women’s ovarian tissue when suffering from PCOS, and are associated with ovulation failure, infertility, and implantation disorders in PCOS patients [43]. The decrease of AA content in BAT transplantation group may be helpful to improve the progress of PCOS. By deciphering the inflammatory responses and oxidative stress alterations along with secretion changes of KGN cells, our findings offer a new understanding of how AA itself contributes to damage in women with PCOS.

## Conclusions

**Fig. 8:**
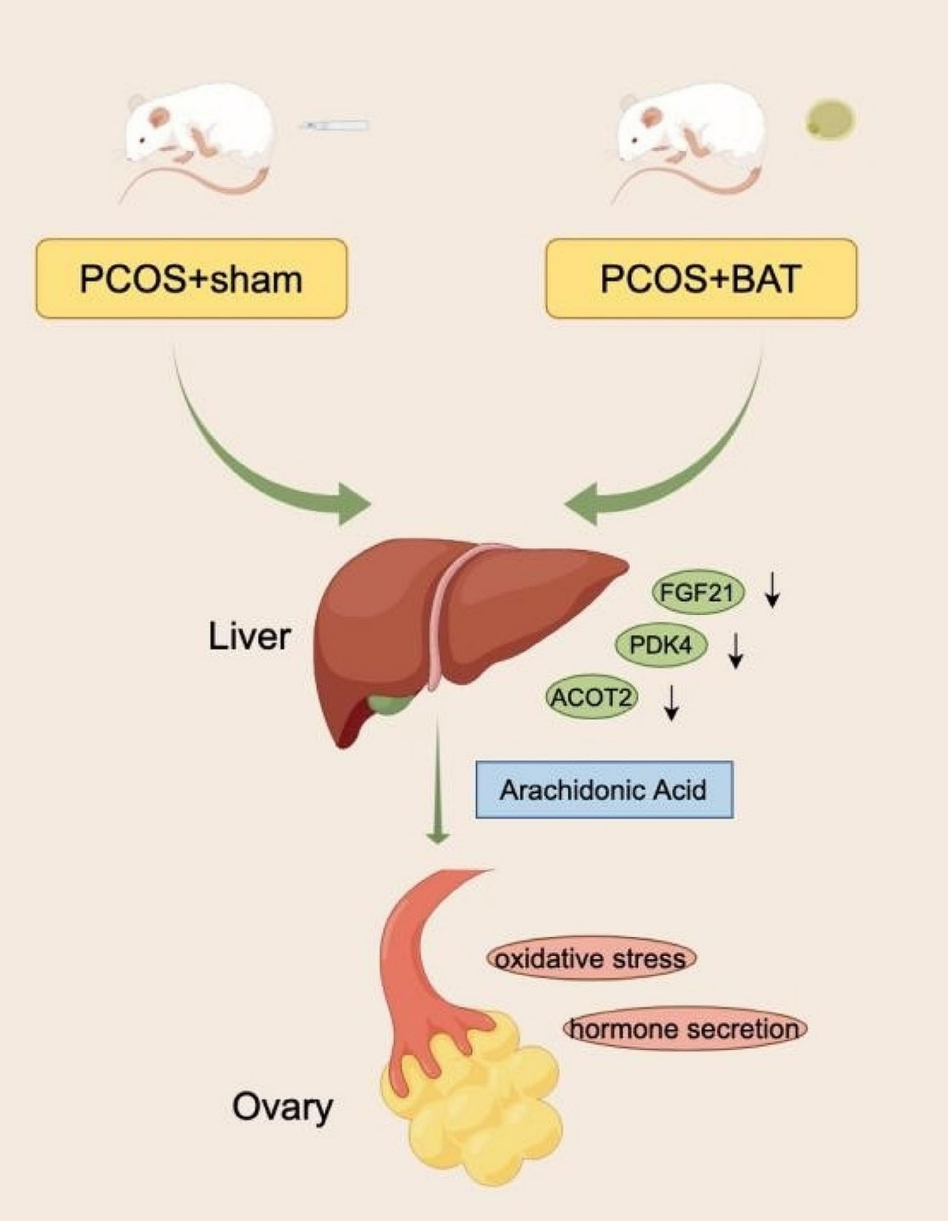
Overview of arachidonic acid inhibit granulosa cell function by affecting metabolic function of liver in Brown adipose transplantation Rats. BAT successfully recovered the reproductive and metabolic phenotype of PCOS rats. The expression of liver factors were improved. Differentially expressed metabolites were analyzed, among them, arachidonic acid may play a role in inhibiting cell proliferation, enhancing oxidative stress reaction, promoting estrogen expression, and reducing progesterone level

In our study, the abnormal estrous cycle, hyperglycemia, the expression of some liver factors was improved after BAT transplantation. As a differential expression metabolite that aggravates PCOS, AA decreased in PCOS + BAT group. AA may play a role in inhibiting cell proliferation, which was enriched in PCOS rats, enhancing oxidative stress reaction, promoting estrogen expression, and reducing progesterone level in KGN cells. Our results suggest that BAT transplantation may be a therapeutic strategy for PCOS. Notwithstanding the mechanism of the effect of AA on KGN cells needs to be further studied, differential expressed metabolites might be crucially important for the role of KGN cells in the pathogenesis of PCOS. Our findings highlight that AA is associated with the pathogenesis of PCOS, and targeted treatment may be a promising therapeutic option for PCOS (Fig. 8).

## Methods

### Animals and tissue transplantation

Ten-week-old female Sprague-Dawley rats (270–300 g) were randomly divided into 2 groups, the control (CTL) group was treated with subcutaneous injection of phosphate buffered saline (PBS) and the PCOS group were injected with DHEA (Cat. No. SJ-HS0488) for 21 consecutive days (6 mg/100 g rat body weight) to induce PCOS. The PCOS rats were randomly divided into two groups: sham-operated (PCOS + sham) group and BAT transplantation (PCOS + BAT) group. The transplanted BAT was derived from the scapular region of 2-week-old normal rats. After intraperitoneal anesthesia, BAT was transplanted subcutaneously to the recipient rat’s dorsal region as soon as possible. The sham-operated rats underwent the same procedure, except receiving donor tissues. All operated rats and CTL group were continued to be fed, estrous cycle was observed, glucose tolerance test (GTT) and insulin tolerance test (ITT) were detected. BAT, liver and ovary tissue were collected after sacrificing the rats. All animals received humane care during the study protocol and during euthanasia.

### GTT and ITT

For GTT, the rats were fasted for 16 h of the next day with free access to drinking water. The blood glucose was recorded before the mice were intraperitoneally injected with 10% D-glucose (1.0 g/kg body weight). Blood glucose levels were measured at 15, 30, 60, 90, and 120 min with Sannuo blood glucose meter (Sinocare Inc. China). For ITT, the mice were subjected 3 days later. The mice were fasted for 4 h with free access to drinking water and then intraperitoneally injected with insulin (1 U/kg body weight; Humulin; Eli Lilly, Indianapolis, IN, United States). Blood glucose levels were measured as described above.

### Estrus cycle detection

Stages of the estrous cycle were confirmed by vaginal smears 11 days following the operation. Rats were recorded as diestrus, proestrus, estrus and metestrus.

### Hematoxylin and eosin staining

The ovary and liver tissue were collected and fixed in 10% neutral-buffered formalin overnight at 25 °C, and dehydrated respectively in alcohol and xylene. Then dehydrated samples were embedded in paraffin and tissue was cut to produce longitudinal sections. After washing 3 times with PBS, the sections were stained with hematoxylin for 2 min and eosin 4 min separately at 25 °C. Morphological changes were subsequently viewed under a light microscope (Nikon, Japan).

### Immunohistochemical staining

The tissues were dehydrated in graded ethanol and transfer to xylene embedded in paraffin after. After the slices were microwaved in a citrate-phosphate buffer (pH 6.0) to retrieve antigens, they were treated with 3% hydrogen peroxide followed by 10% normal goat serum blocking at room temperature for 30 min. Then, the slices were incubated with primary antibodies of UCP-1 (Cat No.AF8292, Beyotime, China) diluted in PBS for 24 h at 4 °C. The sections were incubated with secondary antibodies for 30 min at room temperature following several washes in PBS. Finally, the signals were detected using a biotin-streptomycin-hydroxide system. Negative controls were performed with the primary antibodies replaced by PBS. Morphological changes were subsequently viewed under a light microscope (Nikon, Japan).

### Cell line culture

Asteroidogenic human granulosa-like tumor cell line (KGN cell line) was grown in DMEM (HyClone, USA) supplemented with 10% fetal bovine serum (FBS, HyClone, USA) and 100 U/mL penicillin G and 0.1 mg/mL streptomycin sulfate (HyClone, USA).

### RT-qPCR

Gene expression of liver tissues by using qPCR. Total RNA was isolated from tissue and homogenized in TRIzol reagent (Invitrogen, USA) according to the manufacturer’s instructions and assessed for quantity and purity by using a NanoDrop ND-1000 spectrophotometer (NanoDrop, USA). cDNAs were synthesized by using the reverse transcription kit (Takara, China). Subsequently, based on the literature, qPCR was performed on a StepOnePlus real-time PCR system (Thermo Fisher Scientific, USA) by using SYBR Green PCR master mix (Thermo Fisher Scientific, USA) with primers: 50℃ for 2 min, 95℃ for 2 min, 40 cycles of 95℃ for 15 s and 60℃ for 60 s. GAPDH served as the internal control for gene expression normalization. The relative expression levels of the related genes were calculated using the 2^−ΔΔCT^ method.

The primers are depicted in Table 2.

**Table 2:** List of primers

Gene	Sequence	Sequence (5’-3’)
FGF21	F	AGATCAGGGAGGACGGAACA
FGF21	R	TCAGGATCAAAGTGAGGCGAT
PDK4	F	AACCCTACGGATCCTAGCCA
PDK4	R	GGCATTTTCTGAACCGAAGTCC
ACOT2	F	AACTACGACGACCTCCCCAA
ACOT2	R	CTGGTCCTTTTACCTCAGGGT
GAPDH	F	CTCTGCTCCTCCCTGTTCTA
GAPDH	R	GATACGGCCAAATCCGTTCAC

(F represents forward primer, R represents reverse primer)

### Detection of metabolic profiling by LC-MS

Ultra-High Performance Liquid chromatography (Ultimate 3000, USA) combined with the thermo-Orbitrap Elite mass spectrometer was utilized for the LC-MS analysis. The system was equipped with an electrospray ionizationsource and operated in either positive or negative ionizationmode using a mass resolution of 70, 000 at an m/z of 200. Data-dependent (dd-MS2, TopN = 10) MS/MS mode with a full scan mass resolution of 17, 500 at an m/z of 200 was used. The scan range was 100-1, 500. Metabolic profiles in electrospray ionization (ESI) positive and ESI negative ion modes were performed using an ACQUITY UPLC I-Class system (Waters Corporation, USA) coupled with an AB SCIEX Triple TOF 5600 System (AB SCIEX, USA). The binary gradient elution systems consisted of water containing 0.1% formic acid, v/v (A), and acetonitrile containing 0.1% formic acid, v/v, (B). 20% B for 2 min; 60% B for 4 min; 100% B for 11 min; 100% B for 13 min % B for 13.5 min and finally, 5% B for 14.5 min. The chromatographic conditions were as follows: injection volume was 2 µl; column temperature was 25 °C; flowrate was 0.35 ml/min. Data were acquired in centroid mode using the Thermo Excalibur 2.2 software (Thermo Fisher Scientific, USA).

### Cell viability assay (CCK-8 assay)

The cell viability was detected by Cell Counting Kit-8 (CCK-8) (Kumamoto, Japan). The cells were exposed to AA at the doses of 10µM, 20µM, 50µM and 100µM. After treatment, 10 µl of CCK-8 solution was added to each well, and the 96-well plate was continuously incubated at 37 ℃ for 1 h, then the OD value for each well was read with a wavelength of 450 nm to determine the cell viability on a microplate reader (Multiskan, Thermo Fisher Scientific, USA). The assay was repeated five times. The cell viability was calculated as following:

$$cell{\text{ }}viability{\text{ }}\left( \% \right) = \frac{{OD\left( {\exp eriment} \right) - OD\left( {blank} \right)}}{{OD\left( {control} \right) - OD\left( {blank} \right)}} \times 100$$

### Flow cytometry

KGN cells were collected, washed twice with cold PBS, and then stained with FITC and PI (Beyotime Biotechnology) for 20 min in the dark. Apoptotic cells were analyzed using flow cytometry and FlowJo software (BD Biosciences, USA).

### Detection of ATP/SOD/GPx/MDA

The ATP levels of KGN cells were determined by using ATP assay kit (Beyotime, China) based on the luciferin-luciferase reaction. The KGN cells were lysed and centrifuged at 12,000 g, 4 °C for 5 min. 20 µl supernatants were then mixed with 100 µl detection working solution in a black 96-well plate. Then, the chemiluminescence was measured. The SOD activity was determined by pyrogallol method (420 nm method). The test kit was purchased from Beyotime China. The ATP levels of KGN cells were determined by GSH peroxidase (GPx) assay kit (Beyotime, Chins). 180 µl GSH-PX buffer, 5 µl of diluted sample, 11 µl working solution and 4 µl 15 mM superoxide were added into a 96-well plate and incubated for 20 min at 25 °C, and the absorbance at 340 nm was determined. For the detection of SOD, 20 µl diluted sample were incubated with 160 µl NBT solution and 20 µl reaction regent at 25 °C for 30 min, and the absorbance at 560 nm was read. The MDA content was detected by using MDA assay kit (Beyotime, China). Insoluble materials and proteins were removed from homogenized tissues by centrifugation at 13,000 g at 4 °C for 10 min; 150 µl water containing 3 µl butylated hydroxytoluene (100X) and 2 N perchloric acid was then added to the tissues and vortexed at 25 °C. Next, 200 µl supernatant was mixed with 600 µl thiobarbituric acid solution and incubated at 95 °C for 60 min. Subsequently, 200 µl reaction mixture were added into a 96-well plate and the absorbance at 532 nm was determined by a microplate reader.

### Hormonal assays

Estradiol, progesterone and testosterone concentrations were measured by corresponding ELISA kits (Cat No.PE223, Cat No.PP773 and Cat No.PT872, Beyotime, China).

### Statistical analysis

The metabolic data were acquired using the Thermo Xcalibur 2.2 software (Thermo Fisher Scientific, USA). Peak alignment and extraction wereH performed using the Compound Discoverer software (Thermo Fisher Scientific, USA). The edited data matrix was imported into the SIMCA-P 11.0 software (Umetrics, Sweden) for multivariate statistical analysis, principal components analysis (PCA), and partial least squares discrimination analysis (PLS-DA). The unsupervised PCA analysis assessed the overall trend of segregation between the samples, while a supervised PLS-DA analysis model screened for significantly different metabolites between the PTB and control groups. The ion peaks were normalized and Pareto-scaled. According to PLS-DA model, the variables with variable importance in the projection (VIP) value > 1.0 were selected. Bonferroni correction was used for multiple testing adjustment. In order to identify these potential biomarkers, the accurate ion mass was input into the human metabolome database (HMDB, https://hmdb.ca) databases to match the exact molecular weight, and MS1/MS2 fragment ions were automatically searched. Finally, in order to confirm the structure of the compound, we used our internal standard metabolite library, matching the exact mass, fragment ion mass, and retention time. The ingenuity pathway analysis (IPA) from the Kyoto Encyclopedia of Genes and Genomes (KEGG) online database was applied to understand the functions and interactions of genes and metabolites.

Data were presented as means ± SEM. Statistical analyses were performed by the SPSS Statistics 22.0 software (IBM, USA) and GraphPad Prism 9.0 software (GraphPad Software Inc, USA). Statistical significance was determined according to the sample distribution and homogeneity of variance, while statistical comparisons between two groups were determined by the t-test.

## Data Availability

The datasets used and/or analyzed during the current study are available from the corresponding author on reasonable request.

## Abbreviations

PCOS: polycystic ovary syndrome
BAT: Brown adipose tissue
FGF: Fibroblast growth factor
PDK: Pyruvate dehydrogenase kinase
ACOTs: Acyl-CoA thioesterases
KGN cell: Human ovarian granulosa tumor cell
GTT: Glucose tolerance test
ITT: Insulin tolerance test
UCP-1: Uncoupling protein-1
DHEA: Dehydroepiandrosterone
CTL: Control
PCR: Polymerase chain reaction
LC-MS: Liquid chromatograph mass spectrometer
GPx: GSH peroxidase
SOD: Superoxide dismutase
OS: Oxidative stress
